# Bi-Module Sensing Device to *In Situ* Quantitatively Detect Hydrogen Peroxide Released from Migrating Tumor Cells

**DOI:** 10.1371/journal.pone.0127610

**Published:** 2015-06-02

**Authors:** Ling Yu, YunLi Tian, AnXiu Gao, ZhuanZhuan Shi, YingShuai Liu, ChangMing Li

**Affiliations:** 1 Institute for Clean energy & Advanced Materials, Faculty of Materials & Energy, Southwest University, Chongqing 400715, China; 2 Chongqing Key Laboratory for Advanced Materials and Technologies of Clean Energies, Chongqing 400715, China; 3 Chongqing Engineering Research Center for Rapid diagnosis of Fatal Diseases, Chongqing 400715, China; North Carolina A&T State University, UNITED STATES

## Abstract

Cell migration is one of the key cell functions in physiological and pathological processes, especially in tumor metastasis. However, it is not feasible to monitor the important biochemical molecules produced during cell migrations *in situ* by conventional cell migration assays. Herein, for the first time a device containing both electrochemical sensing and trans-well cell migration modules was fabricated to sensitively quantify biochemical molecules released from the cell migration process *in situ*. The fully assembled device with a multi-wall carbon nanotube/graphene/MnO_2_ nanocomposite functionalized electrode was able to successfully characterize hydrogen peroxide (H_2_O_2_) production from melanoma A375 cells, larynx carcinoma HEp-2 cells and liver cancer Hep G2 under serum established chemotaxis. The maximum concentration of H_2_O_2_ produced from A375, HEp-2 and Hep G2 in chemotaxis was 130±1.3 nM, 70±0.7 nM and 63±0.7 nM, respectively. While the time required reaching the summit of H_2_O_2_ production was 3.0, 4.0 and 1.5 h for A375, HEp-2 and Hep G2, respectively. By staining the polycarbonate micropore membrane disassembled from the device, we found that the average migration rate of the A375, HEp-2 and Hep G2 cells were 98±6%, 38±4% and 32 ±3%, respectively. The novel bi-module cell migration platform enables *in situ* investigation of cell secretion and cell function simultaneously, highlighting its potential for characterizing cell motility through monitoring H_2_O_2_ production on rare samples and for identifying underlying mechanisms of cell migration.

## Introduction

Cell migration plays a role in many physiological and pathological processes, including tumor metastasis.[1–3] It is a physical and chemical multistep cycle including extension of a protrusion, formation of stable attachments near the leading edge of the protrusion, translocation of the cell body forward, and release of adhesions and retraction at the cell rear.[4–6] Cell migration is a prerequisite step for tumor cell invasion and metastasis that is among the most complicated and major pathologic process responsible for metastasis and poor prognosis of cancer patients.[7–9] Based on a western-blot assay, activation of multiple signalling pathways, such as extracellular signal-regulated kinase (ERK), integrin and focal adhesion kinase (FAK), are associated with cell migration.[5, 10–14] Recently, studies have shown that reactive oxygen species (ROS), particularly hydrogen peroxide (H_2_O_2_), diffusing freely through cellular membranes, can function as a signal messenger delivering information between signalling pathways and can even facilitate communication between cells.[15–23] Usatyuk *et al*. reported that ROS generation is responsible for hepatocyte growth factor (HGF) activated c-Met/PI3K/AKT signalling, which is an important pathway linked to cell migration.[16] Other cellular factors like Arp2/3 complex and FAK required for cell migration are also under the control of ROS.[11, 16, 17, 24]Apart from activation of signalling proteins, the influence of H_2_O_2_ on cell migration has been investigated through characterization of cell migration capability under exogenous H_2_O_2_ simulation.[21, 22, 25–27] Polytarchou *et al*. reported that exogenous H_2_O_2_ at a concentration of 5 μM induced human prostate adenocarcinoma LNCaP cell migration in a micro-chemotaxis chamber assay.[21] Luanpitong *et al*. evaluated the impact of various known inhibitors and donors of ROS on cell migration. Their results demonstrated that H_2_O_2_ (100 μM) inhibited lung carcinoma H460 cell migration and invasion in a wound healing assay.[22] This contradiction in results obtained from these studies may be rooted on the dosage of exogenous H_2_O_2_, production site, as well as the tissue type of cells. The next concern is that extremely high, non-physiological concentrations of H_2_O_2_ were used, barely mimicking living cell environment. More importantly is that little is known about endogenous H_2_O_2_ levels during cell migration. In addition, from a methodology point of view, the dominant technique for characterization of cellular ROS is based on probe-labelling assays. Fluorescent histochemistry[28], flow cytometery[29] and spectrofluorimetric analysis[30] are the most widely used approaches to characterize ROS by using fluorescent dyes 2',7'-dichlorofluorescein diacetate (DCFH-DA), hydroethidine (HE) and dihydrorhodamine 123 (DHR) *etc*. These probe-labelling approaches are based on indirect methods that have been shown to be time consuming, difficult to automate and highly prone to interferences.[31] Most importantly, it is not feasible to conduct measurements *in situ* that can provide cell metabolism information and it is not feasible for characterization of cell morphology, not to mention biological functions, such as migration.[32] On the other hand, wound healing assays, trans-well assays or Boyden chamber assays, are widely used for cell migration experiments; however, they are used solely to characterize cell motility by quantifying the number of migrated cells, lacking the capability to probe biochemical changes during migration. Apart from investigation of the impact of exogenous H_2_O_2_ on cell migration, less attention has been paid to directly address H_2_O_2_ production during cell migration or invasion. Therefore, the aim of this study is to define a rational strategy enabling *in situ* monitoring of biochemical changes during the cell migration process for delineating the underlying molecular mechanisms.

Electrochemical sensors demonstrate their potential to analyse cell-secreted biomolecules.[33–36] Dr. McConnell and co-worker investigated extracellular menadiol redox activity by means of an extracellular solution containing the ferricyanide/ferrocyanide couple and a gold electrode.[37] Cytosensor microphysiometer was modified for the electrochemical detection of extracellular acidification, oxygen consumption rates or insulin.[38–40] In our previous study, as low as a 40 μL sample volume was required to probe H_2_O_2_ secreted from tumor cells.[41] The use of a small volume sample allows expensive reagents, particularly for rare clinical biopsies, to be conserved and makes using this analysis more cost-effective. On the other hand, the progress in lab-on-a-chip technology facilitates the study of cellular behaviour under tightly controlled microenvironments with high spatiotemporal resolution.[42–47] Previous endeavours have focused on establishing a microenvironment that mimics *in vivo* conditions for cell migration and analysis of migration at a single cell level.[9, 42–44] But, those achievements mainly illustrated the morphology and functional changes of cells during cell migration. No studies have been reported to study biochemical molecule generation during the cell migration process.

In this work, an electrochemical sensor embedded poly(dimethylsiloxane) (PDMS) device was developed to monitor H_2_O_2_ *in situ* during tumor cell migration process. To achieve this goal, a multi-wall carbon nanotube (MWCNT)/graphene/MnO_2_ composite functionalized indium tin oxide (ITO) glass electrode was fabricated as a H_2_O_2_ sensing module. This H_2_O_2_ sensing module was assembled with a cell migration module that is a PDMS chamber/polycarbonate membrane/PDMS chamber sandwich structure. The fully assembled bi-module device *in situ* sensed H_2_O_2_ production of human melanoma cell migration under a serum established chemotaxis field. The effect of the cell H_2_O_2_ production inhibitor, diphenyleneiodonium (DPI), and H_2_O_2_ decomposition enzyme, catalase, on cell migration was also investigated on assembled devices. H_2_O_2_ generation and migration capability measured with assembled devices were interpreted with standard Boyden transwell assays and the results confirmed that the fully assembled bi-module device could indeed monitor H_2_O_2_ *in situ* during cell migration.

## Materials and Methods

### Materials

Graphite, multi-walled carbon nanotubes (MWCNT), ascorbic acid, 30% hydrogen peroxide, potassium hexacyanoferrate (III) (K_3_[Fe(CN)_6_]), Nafion were purchased from Aladdin, China. Phosphate buffered saline (PBS), potassium permanganate (KMnO_4_) were from Chongqing co. Indium tin oxide (ITO) glass and silver paste were obtained from Jieshen Electronics Technology CO. Ltd (China). Printed circuit broad (PCB) UV photosensitive dry film (40 μm) was obtained from IC Machinery Equipment Group (China). Human melanoma cells, A375, were obtained from ATCC. Human liver carcinoma cell line Hep G2 and human Larynx carcinoma cell line HEp-2, gifts from Dr. Yuan Li (Chongqing Medical University), were originally purchased from China Center for Type Culture Collection. The cells were maintained in RPMI 1640 medium (Gibco) with 10% fetal bovine serum (Gibco), 100 μg mL^-1^ penicillin and 100 μg mL^-1^ streptomycin. Phorbol 12-myristate-13-acetate (PMA), di-phenyleneiodonium (DPI), catalase and Whatman Cyclopore polycarbonatemembrane (cyclopore PC circles, 5.0 μm) were purchased from Sigma Aldrich. PMA and DPI were dissolved with dimethyl sulfoxide (DMSO) to a concentration of 5 mg mL^-1^ and 10 mM, respectively, as a stock solution. All other chemicals used in this study were analytical grade. The deionized (DI) water used in all experiments was produced by a Q-Grad1 system, Millipore Corporation.

### Bi-module device design and fabrication

**Fig 1:**
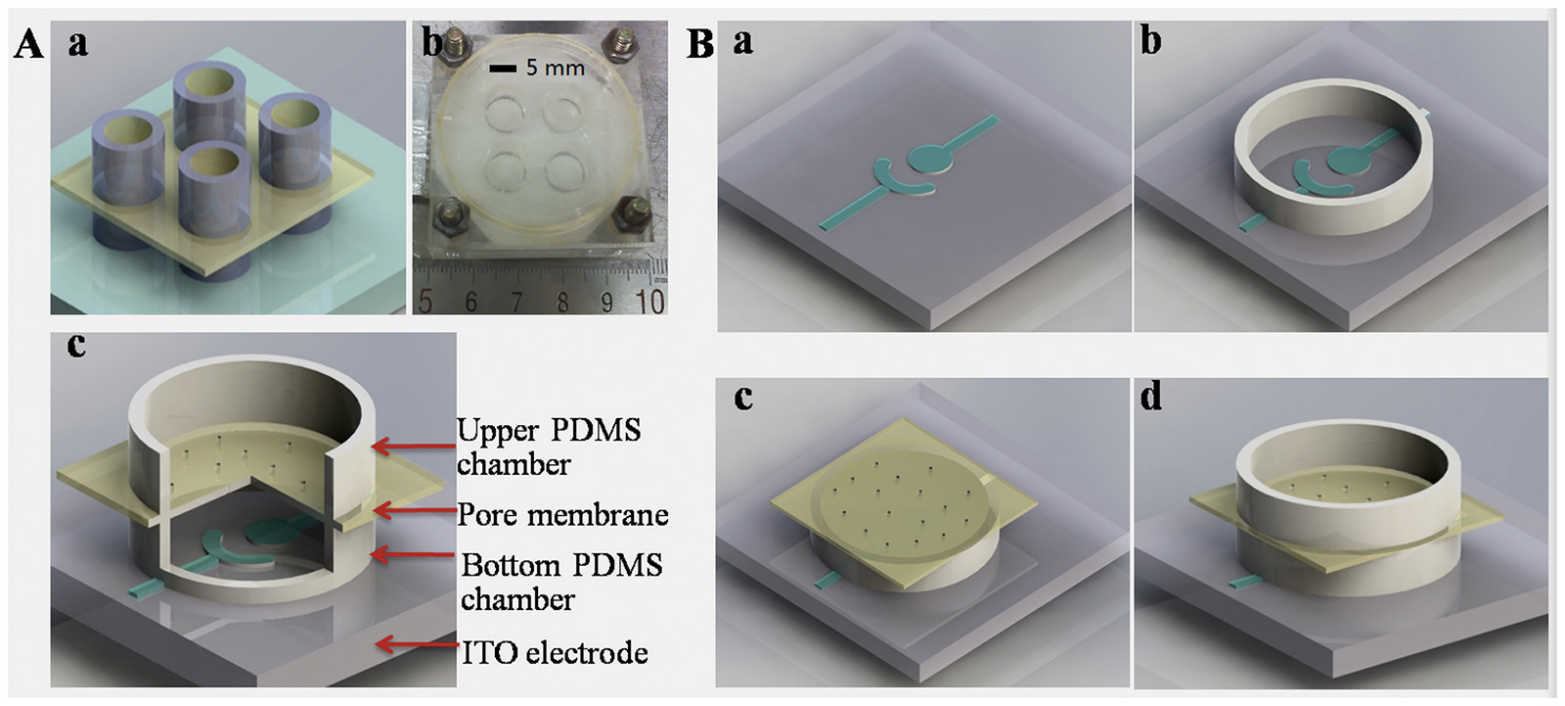
(A) 3D image of the bi-module device that consists of an electrochemical detection module and cell migration module (*a*); photograph of a device for experiment (*b*); section view of a bi-module devise (*c*); (B) Schematic diagram of the micro-fabrication processes: patterning and fabrication of electrodes on a ITO glass (*a*); bonding of a PDMS ring (5 mm in diameter, 1 mm in height) with ITO glass (*b*); assemble of a polycarbonate membrane on top of the PDMS right (*c*); assemble of a PDMS ring (5 mm in diameter, 5 mm in height) on top of the membrane (*d*). ITO: indium tin oxide, PDMS: Poly(dimethylsiloxane).

As depicted in Fig 1A, the device consists of two modules: an electrochemical sensor for H_2_O_2_ detection and a micro trans-well platform for cell migration. From bottom to top, there are an electrochemical sensor, a PDMS chamber, a polycarbonate micropore membrane and another PDMS chamber. The micropores on the polycarbonate membrane are the channel for cells to transport from the upper chamber to the bottom chamber. Fig 1B describes the schematic diagram of the micro-fabrication process for the bi-module device. A two-electrode system was chosen to build the electrochemical sensor. The size of the working electrode and reference/counter electrode is 4.91 mm^2^ and 12.8 mm^2^, respectively. To fabricate electrodes using ITO glass, a dry photosensitive film (40 μm) was coated on the ITO glass and patterned following a UV photolithography process. ITO layer that was not covered by photosensitive film was dissolved by immersing the chips in etchant solution (37%HCl: H_2_O:FeCl_3_•6H_2_O = 3L:1L:25g) for 30min. Finally, the patterned electrodes were recovered by removing the residue photosensitive film (***a***). A PDMS ring with a diameter of 5 mm and a height of 1mm was treated by plasma cleaner (Harrick, PDC-002) for 60 second and then bonded with ITO electrodes (***b***). To assemble a transwell chamber for assaying cell migration, a polycarbonate membrane was placed on top of the PDMS ring (***c***). Finally, another PDMS ring with a diameter of 5 mm and height of 5 mm was assembled on top of the polycarbonate membrane (***d***).

### Electrochemical device for hydrogen peroxide analysis

The sensing material for hydrogen peroxide (H_2_O_2_) detection used in this study was a MWCNT/graphene/MnO_2_ aerogel. This functional material was synthesized according to our previous study.[41] In brief, a mixture containing MWCNT (1mg mL^-1^), graphene oxide (1mg mL^-1^) and KMnO_4_ (10mg mL^-1^) was prepared and stirred at room temperature for 16 h. Then, the reaction mixture was centrifuged to collect the precipitate. Next, the re-suspended precipitate was mixed with ascorbic acids solution (100 mg mL^-1^) at 50°C for 15h to form a MWCNT/graphene/MnO_2_ hydrogel, and then freeze-dried for 24 h to completely remove water. The obtained aerogel (MWCNT/graphene /MnO_2_) was dispersed in 500 μL of ethanol (5 mg/mL) and casted onto the surface of ITO working electrodes. The MWCNT/graphene/MnO_2_-functionalized electrode was characterized by cyclic voltammetry (CV) in 0.5 M KCl solution containing 50 mM K_3_Fe(CN)_6_ at the scan rate of 10 mVs^-1^. Then the amperometric response of the fully assembled electrochemical sensor to H_2_O_2_ was characterized with RPMI 1640 medium according to the literature.[41] To analyse the stability of the MWCNT/graphene/MnO_2_ functionalized electrode, the electrode was immersed in the cell culture medium for 24 h. The CV response of the electrode was recorded when adding H_2_O_2_ (4 μM) in to the cell culture medium at 0, 12, 18 and 24 h and the changes of reduction peak current of the CV curve was compared.

### Quantification of H2O2 production from migrating cell in a fully assembled device

**Fig 2:**
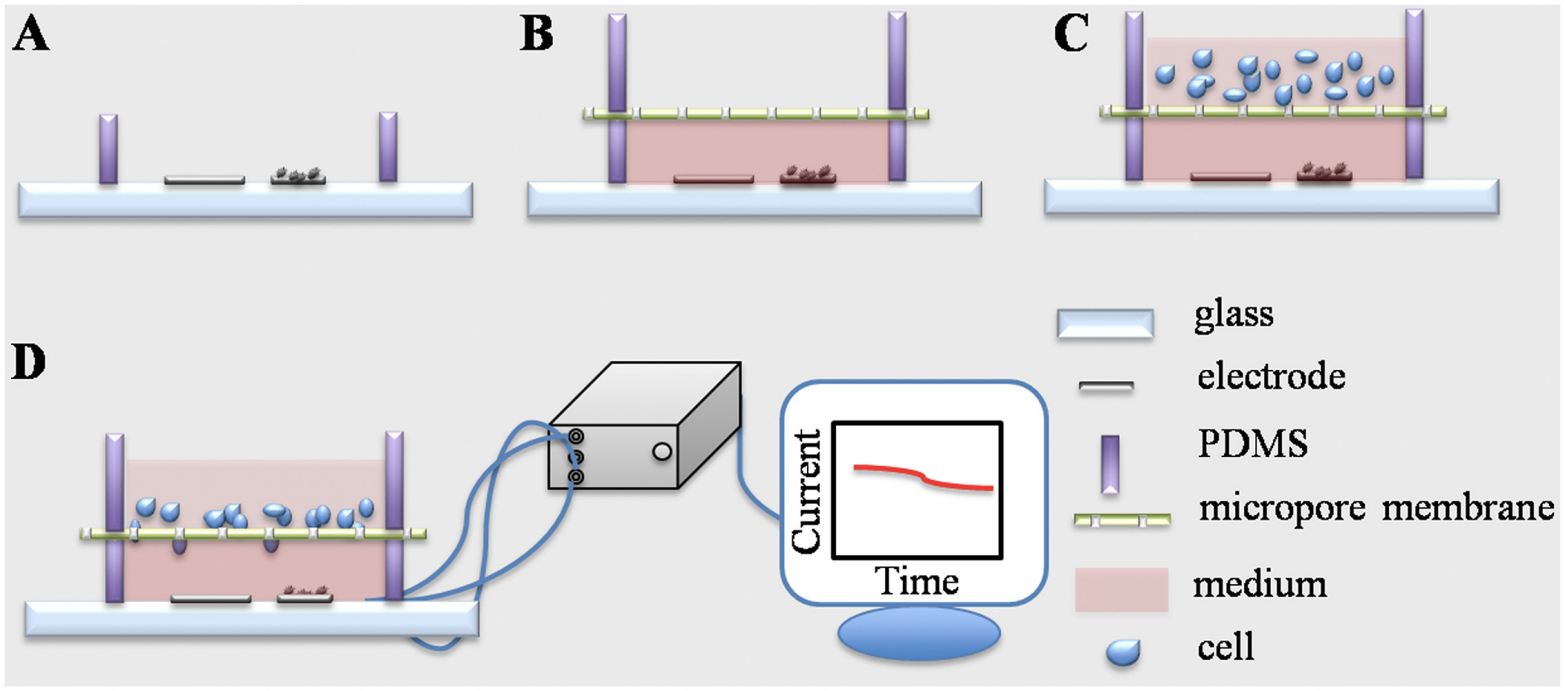
Experimental protocol for the electrochemical detection of H_2_O_2_ production during cell migration. (*a*) MWCNT/graphene/MnO_2_ functional material is casted on working electrode (right one) surface following a layer of Nafion coating; (*b*) RPMI 1640 medium is injected into the PDMS ring (bottom) using a micropipette; then polycarbonate membrane is placed on top of the PDMS ring (bottom), and another piece of PDMS ring (upper) is aligned over the membrane; (*c*) cell suspension is injected into the upper cell seeding chamber using a micropipette; (*d*) the cell-loaded device is incubated in a cell incubator for 12h and the electrochemical signal is recorded. MWCNT: mutil-wall carbon nanotube, PDMS: Poly(dimethylsiloxane).

Human melanoma A375 cells, liver cancer Hep G2 and larynx carcinoma HEp-2 cells were cultured in RPMI 1640 medium supplemented with 10% FCS under standard conditions (37°C, 5% CO_2_). Fig 2 illustrates the measurement settings. A functional sensing material, MWCNT/graphene/MnO_2_ in ethanol (5 mg/mL), was cast on the working electrode. Then one microliter of Nafion that was diluted in ethanol (1:30, V/V) was casted (***a***). 50 μL of RPMI 1640 medium, with or without serum, was placed in the bottom PDMS chamber. A polycarbonate membrane and another PDMS ring were assembled on top of the bottom chamber in order (***b***). Next, serum-starved tumor cells (1×10^6^) in 100 μL serum-free RPMI 1640 medium were placed into the top chamber (***c***). All steps were conducted in a biological hood with caution to avoid microorganism contamination. Finally, the device with cell-loading was placed in a cell culture incubator maintaining stable temperature and CO_2_ atmosphere (37°C, 5% CO_2_). The copper wires were linked to an electrochemical station (CHI 760) and the amperometric signal (*i-t* curve) was recorded for 12 h (***d***). The signal from the device without cell loading was recorded as a basal control. Cells incubated with a H_2_O_2_ generation inhibitor DPI (10 μM) and a H_2_O_2_decomposer catalase (5 μg mL^-1^) were measured in parallel. Since the DPI and PMA were dissolved in DMSO, the impact of this organic solvent (0.5%, V/V) on H_2_O_2_ production was evaluated. After electrochemical measurement, the polycarbonate membrane in the device was disassembled and the migrated cells were visualized by hematoxylin and eosin (H&E) staining. In brief, the membrane was immersed in 4% paraformaldehyde solution for 10 min and then stained by hematoxylin and eosin solution for 10 and 2 min, respectively. Finally, loosely attached cells on topside of the membrane were removed by scrubbing twice with cotton tipped swab.[48] The cell visualized on the bottom side of the membrane was defined as migrating cell. Six randomly selected fields per membrane were imaged (Olympus IX73, Japan) and the number of the purple-stained cells was counted. The percentage of migrated cells was calculated using medium without serum in the bottom chamber as a reference. All experiments were repeated three times independently.

### Statistical analysis

Results are expressed as means ± the standard error of the mean (SEM). The data were analyzed by Student’s *t*-test using Origin Statistic software (OriginLab Corporation, USA). A *p*-value < 0.05 was considered significant.

## Results and Discussion

### Electrochemical characterization of the assembled device

**Fig 3:**
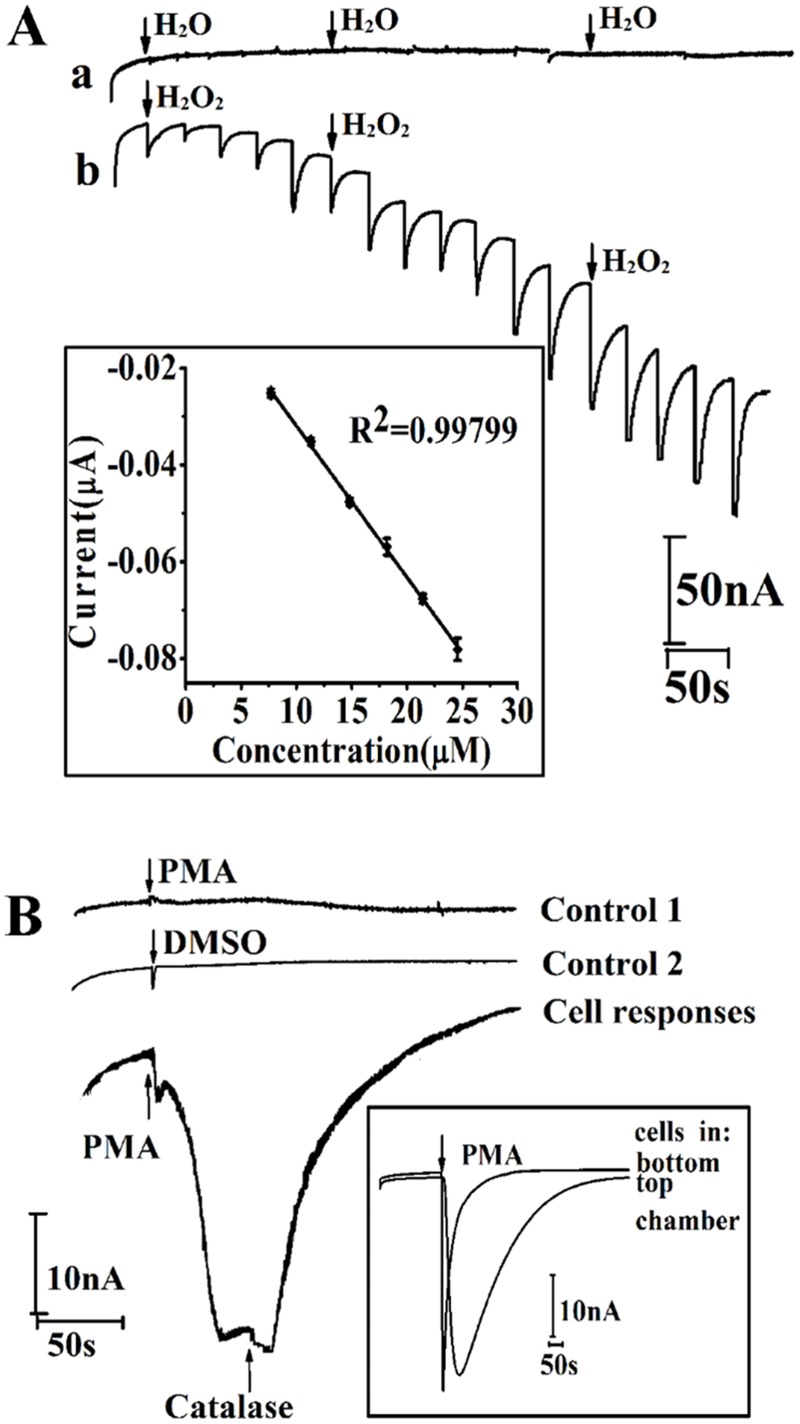
Amperometric performance (*i-t* curve) of fully assembled device. (A) *i*-*t* curves of successive additions of H_2_O (*a*) or 4 μM H_2_O_2_ (*b*) into RPMI 1640 at an applied potential of -0.4 V *vs* ITO RE/CE; (B) *i-t* curves of PMA injection (0.5mg mL^-1^) without cells loading (control 1), DMSO (0.5%, v/v%) injection with cell loading (control 2), PMA injection (0.5 mg mL^-1^) with cells loading, and followed by catalase injection (5 μg mL^-1^)-cell response at an applied potential of -0.4 V *vs* ITO RE/CE; Inset of (B) *i-t* curves of PMA injection with cells loaded in top and bottom chamber of assembled device. RE/CE: reference electrode/ counter electrode; PMA: phorbol 12-myristate-13-acetate, DMSO: dimethyl sulfoxide

Our previous study demonstrated that MWCNT/graphene/MnO_2_ specifically responses to H_2_O_2_.[41] To evaluate the stability of the sensor that immersed in cell culture medium for 24 h, 1 mM H_2_O_2_ was added into the medium at 0, 12, 18 and 24 h, and the CV response was recorded. The H_2_O_2_ induced peak current change (S1 Fig) shows that immersing the MWCNT/graphene/MnO_2_ decorated electrode in cell culture medium for 24 h would not attenuate the function of the sensor. To realize H_2_O_2_ production *in situ*, attention has been paid on the amperometric response of the MWCNT/graphene/MnO_2_ functionalized device to subsequent additions of H_2_O_2_ in cell culture medium (RPMI 1640). First, the choice of the applied potential at the working electrode is optimized to achieve a higher sensitivity. The amperometric *i-t* curves under potentials between -0.3 V and −0.5 V (*vs* ITO reference electrode/counter electrode, RE/CE) were recorded. As shown in supplementary S2 Fig, the best performance is obtained with a potential of -0.4 V *vs* ITO RE/CE. Therefore, the sensitivity of the device was monitored amperometrically at the working potential of −0.4 V *vs* ITO RE/CE. Deionized water (DI H_2_O) and 4 μM H_2_O_2_ were subsequent added in medium. The amperometric response of adding DI water is barely observed; indicating the action of pipetting liquid into the reaction chamber does not spike noise (Fig 3A, curve ***a***). While, the amperometric signal in curve ***b*** of Fig 3A shows that the device responds quickly to the change of H_2_O_2_ concentration. Inset of Fig 3A shows the calibration curve of the fully assembled device for H_2_O_2_ detection with a linear equation of Current (μA) = -5.8×10^–4^–0.00313C_H2O2_ (H_2_O_2_ concentration, μM), of which the R^2^ is 0.997 and standard error of the slope is 6.2×10^–5^. The sensitivity of the fully assembled devices is 3.2 nAμM^−1^cm^−2^, based on the ratio of the slope of current-dose response curve and the surface area of electrode. Finally, human melanoma cells A375 (1×10^5^ cell) were cultured on the upper chamber of the device. The *in situ* monitoring of H_2_O_2_ release was investigated by using phorbol 12-myristate-13-acetate (PMA), a model drug known to trigger H_2_O_2_ production from human cells. Meanwhile, catalase, a H_2_O_2_ scavenger, was measured along with PMA to investigate the specificity of the *in situ* monitoring of H_2_O_2_ secreted from cells in the upper chamber. As presented in Fig 3B, no current response was observed from the device without cells (line: control 1) and the device with cultured cells under DMSO (solvent of PMA) injection (line: control 2). With the addition of catalase that can decompose H_2_O_2_ to water and oxygen, the reduction peak current increase caused by PMA injection decreases sharply (line: cell response). It has been reported that H_2_O_2_ can diffuse through cellular membranes to a distance even nearly 1mm because of its solubility in both lipid and aqueous environments and comparatively low reactivity.[33, 49] To investigate the effect of the cell location on H_2_O_2_ detection, we measured the production of H_2_O_2_ from cells growing in the upper and bottom PDMS chamber of the assembled device. A similar current intensity was observed from cells seeded in upper and bottom chamber upon PMA challenge (inset of Fig 3B), indicating the electrochemical sensor located at the bottom chamber can *in situ* sense H_2_O_2_ secreted from cells seeding in the top chamber.

### Quantification of H_2_O_2_ generation during cell migration by electrochemical devices

**Fig 4:**
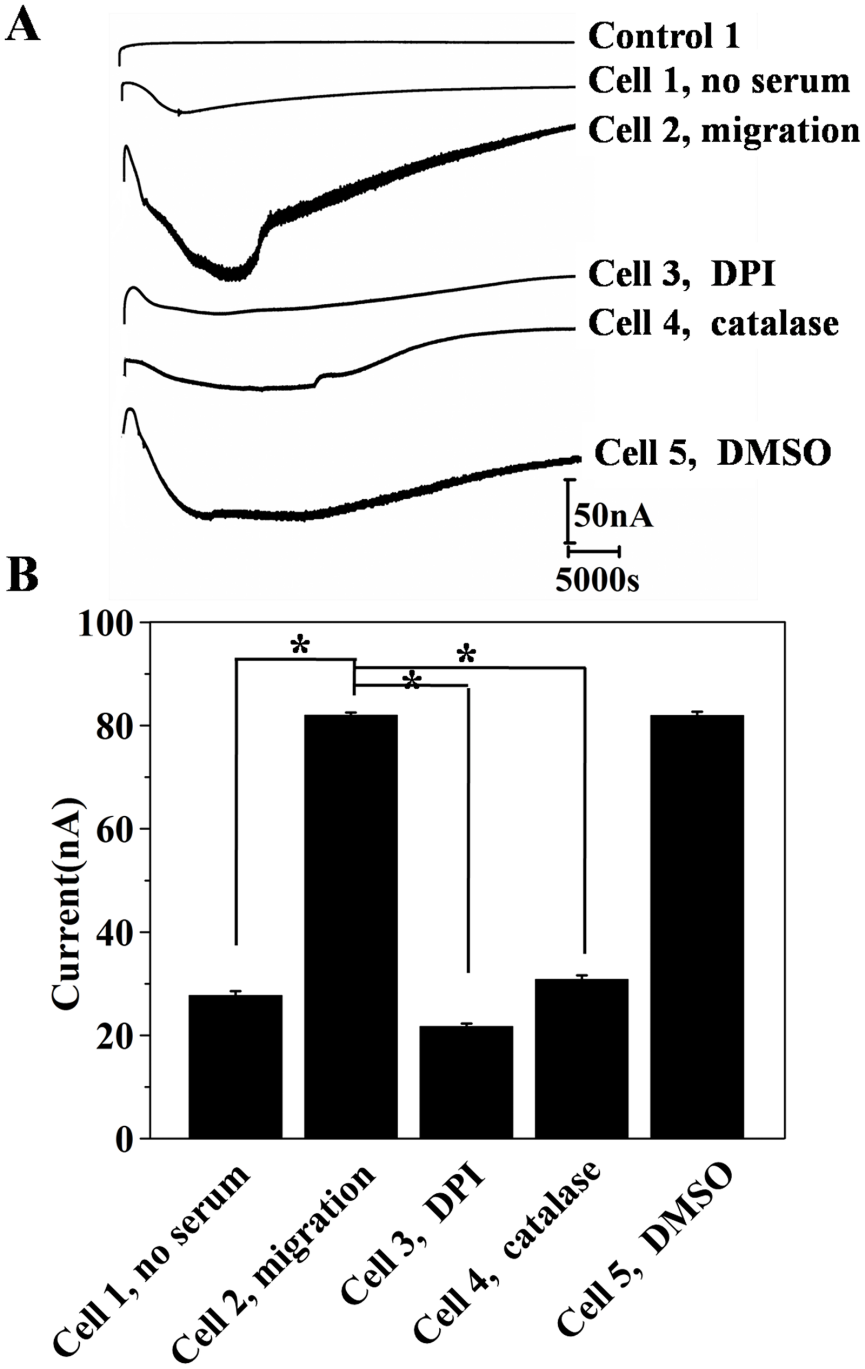
(A) Amperometric responses of fully functionalized bi-module device during melanoma A375 cell migration. *No cell*: device without cell loading; *No serum*: medium without serum in bottom chamber; *DPI*: RPMI 1640 containing 10% serum (conditioned medium) in bottom chamber, cell in upper chamber was incubated with H_2_O_2_ generation inhibitor, DPI; *Catalase*: conditioned medium in bottom chamber, cell in upper chamber was incubated with H_2_O_2_ decomposer, catalase; *Cell migration*: conditioned medium in bottom chamber; *DMSO*: conditioned medium in bottom chamber, cell in upper chamber was incubated with DMSO (solvent of DPI). (B) The corresponding current response obtained from amperometric curves of three independent experiments, (n = 3, * denotes *p*<0.05). DPI: diphenyleneiodonium.

Fig 2 lists experiment settings used in the migration assay with bi-module devices. In cell migration assay, serum-starved (8 h) melanoma A375 cells were studied as the model cell. Fetal bovine serum (FBS) was added to RPMI 1640 medium in the bottom chamber to establish nutrition chemo-attractant. Fig 4A shows the *in-situ* amperometric signal of the melanoma A375 cells that was monitored for 12 h at 37°C. The current baseline of the electrochemical device during a 12 h incubation time was recorded without cell loading (control 1). No visible current change was observed indicating the H_2_O_2_ will not automatically be generated from the medium during 12h incubation. The signal from cells that were seeded in a device with serum free medium in the bottom chamber was characterized as a migration control (cell 1, no serum in top and bottom chamber). The amperometric track shows a current increase (16–28 nA) at the time course of 2–4 h, while the current gradually flows back during 5–7 h and stays stable during the rest of the assay time. We tested the H_2_O_2_ generated from cells seeded in a device in which RPMI 1640 medium plus 10% FBS (conditioned medium) was placed in the bottom chamber. The *in situ* measurement shows a cathode current increase trend and the current change reaches the maximum 84 ±1 nA at 3h. For the rest of the time, the current gradually traced back to baseline (cell 2, migration). To specify that the amperometric signal was indeed given by H_2_O_2_ production during cell migration, NADH oxidase inhibitor DPI (10 μM) and H_2_O_2_ decomposer catalase (5 μg mL^-1^) were used to pre-treat cells loaded in the upper chamber. Cell response 3 is the current signal from DPI pre-treated cells that were seeded in a device containing conditioned medium in the bottom chamber. A maximum current increase (23 ±1 nA) can be read from the *i-t* curve. While, for cells incubated with catalase, a similar *i-t* curve was recorded. The impact of DMSO (solvent of DPI) on H_2_O_2_ productionwas measured in a cell migration section. Fig 4B shows the histogram of current change at a time point of 3h. The highest current change (84±1 nA) is given by cells responding to medium containing 10% serum. The current value obtained from serum-starved cells incubated with DPI and catalase in devices that contained conditional medium (RPMI 1640 plus 10% FBS) in the bottom chamber are 23±1 nA and 32±2 nA, respectively, which are significantly lower than the non-pre-treated cells. Previous studies argued that DPI shows paradoxical effect in inducing DNA damage, mitochondria dysfunctional and even apoptosis.[50–52] To investigate if the small current increase was caused by DPI impaired cell growth, we compared the viability of cells pre-treated by DPI, catalase and DMSO using MTT method (S3 Fig). The results show that DPI (10 μM) or catalase (5 μg mL^-1^) does not reduce the viability of melanoma A375 cell.

In addition, according to the sensitivity of the electrochemical device as characterized in Fig 3A, H_2_O_2_ produced from cells can be calculated as detailed in the literature.[33, 41] At the 3h time point, the generated H_2_O_2_ from none pre-treated, DPI and catalase pre-treated cells are 0.13, 0.034 and 0.049 μM, respectively. Unlike previous fluorescent intensity qualitative descriptions of endogens H_2_O_2_ variation, this is the first time that H_2_O_2_ production during a cell function has been directly quantified. According to the literature[21], exogenous 5 μM H_2_O_2_ enhances tumor cell migration. Thus, we investigated if endogenous H_2_O_2_ of one order lower level (0.1 μM) generated under chemotaxis would associate with the capability of cell migration.

**Fig 5:**
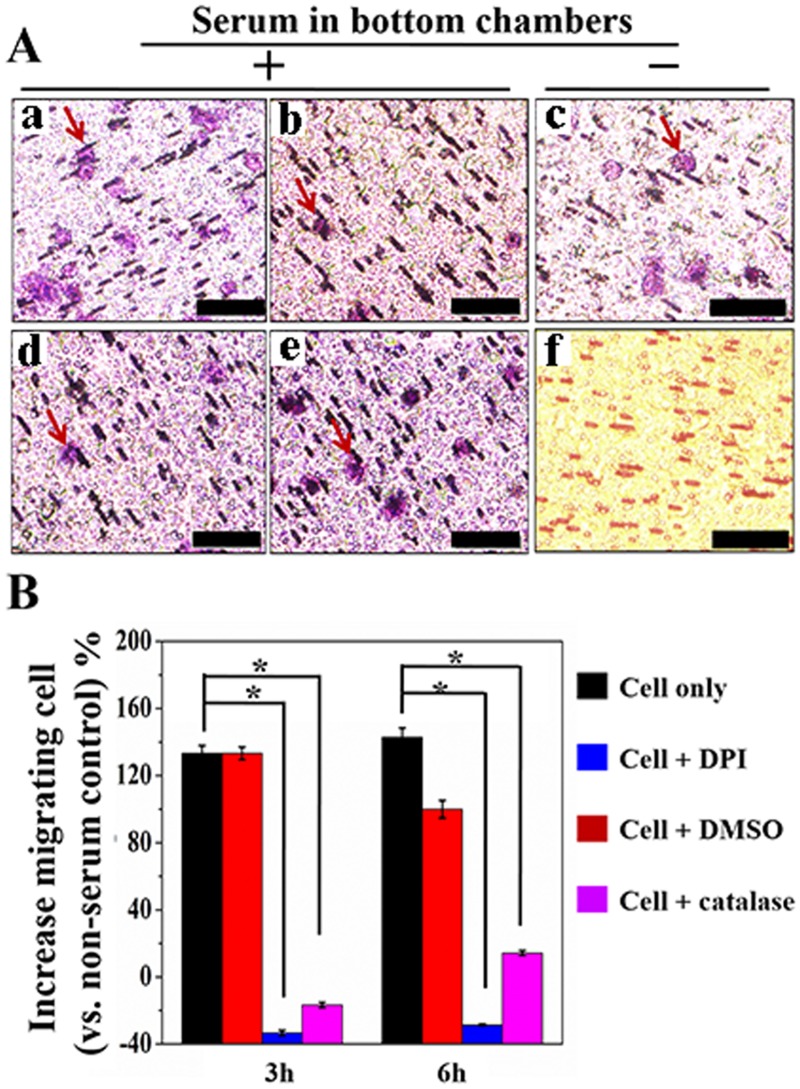
Cell migration quantified by hematoxylin and eosin (H&E) staining the polycarbonate membrane disassembled from device (A) photographs captured under microscopy: RPMI 1640 containing 10% serum in bottom chamber and serum-starved cells in top chamber (*a*), cells pre-treated by DPI (10 μM) (*b*), cells pre-treated by catalase (5 μg mL^-1^) (*d*), cells pre-treated by DMSO (0.5%, V/V) (*e*), serum-starved cells in a device containing serum free medium in top and bottom chambers (*c*), and fresh polycarbonate membrane after (H&E) staining (*f*). Red arrow points the migrating cells. (B) histogram of migrating cell percentage using cells loaded in a device without serum in top and bottom chambers as a reference, * denotes *p*<0.01, n = 3.

To examine if the H_2_O_2_ production is associated with cell migration in the 12 h period, a migration experiment was conducted parallel to quantifying H_2_O_2_ with bi-module devices *in situ*. The polycarbonate membrane disassembled from the device was stained by hematoxylin and eosin (H&E) solution. Fig 5A shows the representative H&E staining images of polycarbonate membranes that was placed on a glass slide. The rod-like objects in all images are the micropore of the membrane. The migrating cells are characterized as purple-staining spot. More cells are observed from polycarbonate membrane that was disassembled from device containing serum-starved cell in the upper chamber and conditioned medium in the bottom chamber. Purple-staining cell sharply reduced on membrane from DPI and catalase pre-treatment groups. By counting the purple-staining cells from six random recorded microscopy images, the migration percentage was calculated using the group with no serum medium in bottom and top chamber as a reference. The results show that serum established chemotaxis induces 133±5% and 143±6% cell migration at 3, and 6 h (Fig 5B). Cells incubated with H_2_O_2_ generation inhibitor DPI can significantly reduce the chemotaxis triggered migration. The catalase pre-treated cells also show less migration (-17±2% and 14±2% cell migrated at time of 3 and 6 h). Collectively, cell migration experiment on the bi-module device showed that serum-starved cells under serum-established chemotaxiscan produce H_2_O_2_, while the production of H_2_O_2_ is associated with cell motility.

Next, a Boyden chamber assay was conducted side-by-side to quantify cell migration. The results demonstrate that cells loaded with different cell densities can develop cell migration under serum-established chemotaxis (S4 Fig). Comparing with standard Boyden chamber assays, wound healing assay and previous reported on-chip cell migration platforms (Table 1), the bi-module device not only capable for study the morphology and functional changes of cells during cell migration, but monitor the generation of H_2_O_2_, an important reactive oxygen species having pathology and physiology significance.

**Table 1:** Performance comparison of bi-module device with standard biological migration assay and lab-on-chip migration assay.

Assay platform	Morph-ology	Biochemical Molecule	Cell function	Chemo-taxis	Ref
**Bi-module device**	YES	YES: Electrochemical analysis of H2O2 generation during migration assay	YES: Migration of cells based on a serum established chemotaxis	YES	***a***
Boyden chamber	YES	NO	YES: Migration of cells into a wound to close the gap	YES	[20, 21]
Wound healing	YES	NO	YES: Migration of cells based on a chemical environment	NO	[19, 25]
On-chip ECIS	NO	NO	YES: Single cell migration	YES	[38]
On-chip wound healing	YES	NO	YES: Cell in responding to either promote or inhibit cell migration	YES	[42, 43]
Lab-on-Chip Mimicking cell surrounding	YES	NO	YES: 3D structure mimic micro-environment *in vivo*.	YES	[39, 41]
**Assay platform**	**Morph-ology**	**Biochemical molecule**	**Cell function**	**Chemo-taxis**	**Ref**
**Bi-module device**	YES	YES: Electrochemical analysis of H2O2 generation during migration assay	YES: Migration of cells based on a serum established chemotaxis	YES	***a***
Boyden chamber	YES	NO	YES: Migration of cells into a wound to close the gap	YES	[20, 21]
Wound healing	YES	NO	YES: Migration of cells based on a chemical environment	NO	[19, 25]
On-chip ECIS	NO	NO	YES: Single cell migration	YES	[38]
On-chip wound healing	YES	NO	YES: Cell in responding to either promote or inhibit cell migration	YES	[42, 43]
Lab-on-Chip Mimicking cell surrounding	YES	NO	YES: 3D structure mimic micro-environment *in vivo*.	YES	[39, 41]

***a***: this work;

H&E: hematoxylin and eosin; ECIS: electrical cell−substrate impedance sensing; 3D: three dimension

**Fig 6:**
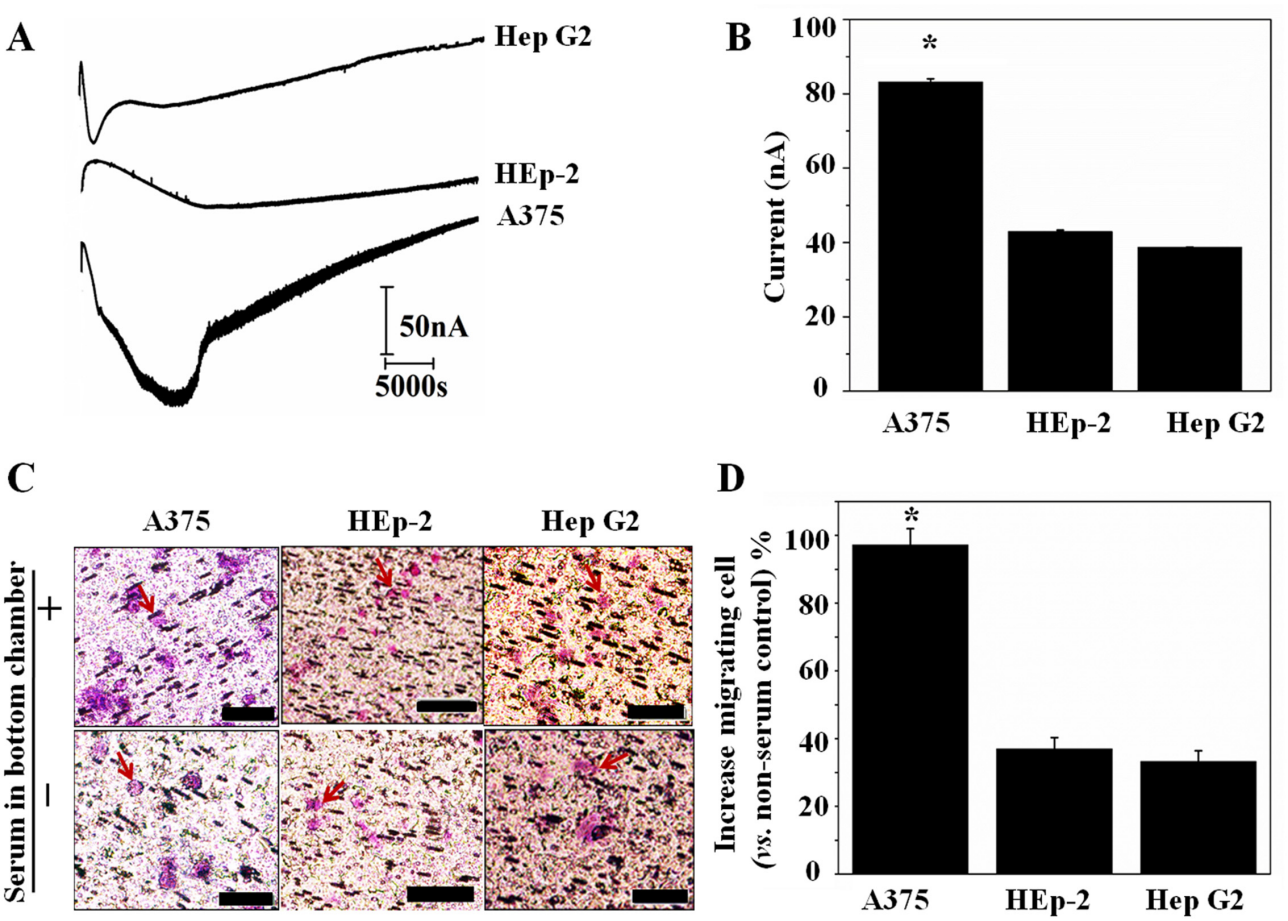
Cell migration quantified by bi-module device. (A) *in situ* amperometric signal of melanoma A375 cell, larynx HEp-2 cell and liver cancer Hep G2 cell seeded in device containing conditional medium (B) histogram of maximum current signal during 12h of cells; (C) H&E staining of migrating A375, Hep G2 and HEp-2 cells; (D) histogram of increased migrating cell percentage using cells loaded in a device without serum in bottom chamber as a reference, * denotes *p*<0.05, n = 3.

Since cell motility is an important factor associated with tumor metastasis, we studied three types of tumor cells with bi-module devices. A375 cells are a well-recognized malignant melanoma cell line. The HEp-2 cell line was originated from tumors, which were produced in irradiated-cortisonised weanling rats after injection of epidermoid carcinoma tissue isolated from the larynx of a male. Hep G2 cells are a human liver carcinoma cell line and a suitable *in vitro* model system for the study of polarized human hepatocytes. Fig 6A presents typical amperometric signal traces of A375, HEp-2 and Hep G2 cells seeded in the upper chamber of bi-module device in which culture medium containing FBS was placed in the bottom chamber. The highest current value can be read at time point of 3.0, 4.0 and 1.5 h from the amperometric trace of A375, HEp-2 and Hep G2, respectively. And the maximum H_2_O_2_ production induced amperometric signal is 84±2 nA for A375 cells, 43±1 nA for HEp-2 cells and 39 ±1 nA for Hep G2 cells (Fig 6B) and the corresponding H_2_O_2_ concentration was 130±1.3 nM, 70±0.7 nM and 63±0.7 nM, respectively. The number of H_2_O_2_ molecule produced per cell is 6.5×10^10^, 3.6×10^10^ and 3.2×10^10^ for A375, HEp-2 and Hep G2 cell, respectively, calculating from the amperometric signal according to literatures.[33,41] Comparing to PMA triggered H_2_O_2_ production[33,41], the H_2_O_2_ molecule produced per cell under serum established chemotaxis is one order smaller. In a parallel experiment, we examined the migrating cells by counting the purple-staining cells on the polycarbonate membrane of the device. As shown in Fig 6C, more A375 cells can be observed from the H&E stained polycarbonate membrane. The average increased migrating cell of A375, HEp-2 and Hep G2 cells after 12 h incubation are 98±7%, 38±4% and 32±3%, respectively (Fig 6D). The quantitative analysis in Fig 6B and 6D confirms that a H_2_O_2_ production corresponds to cell motility.

**Fig 7:**
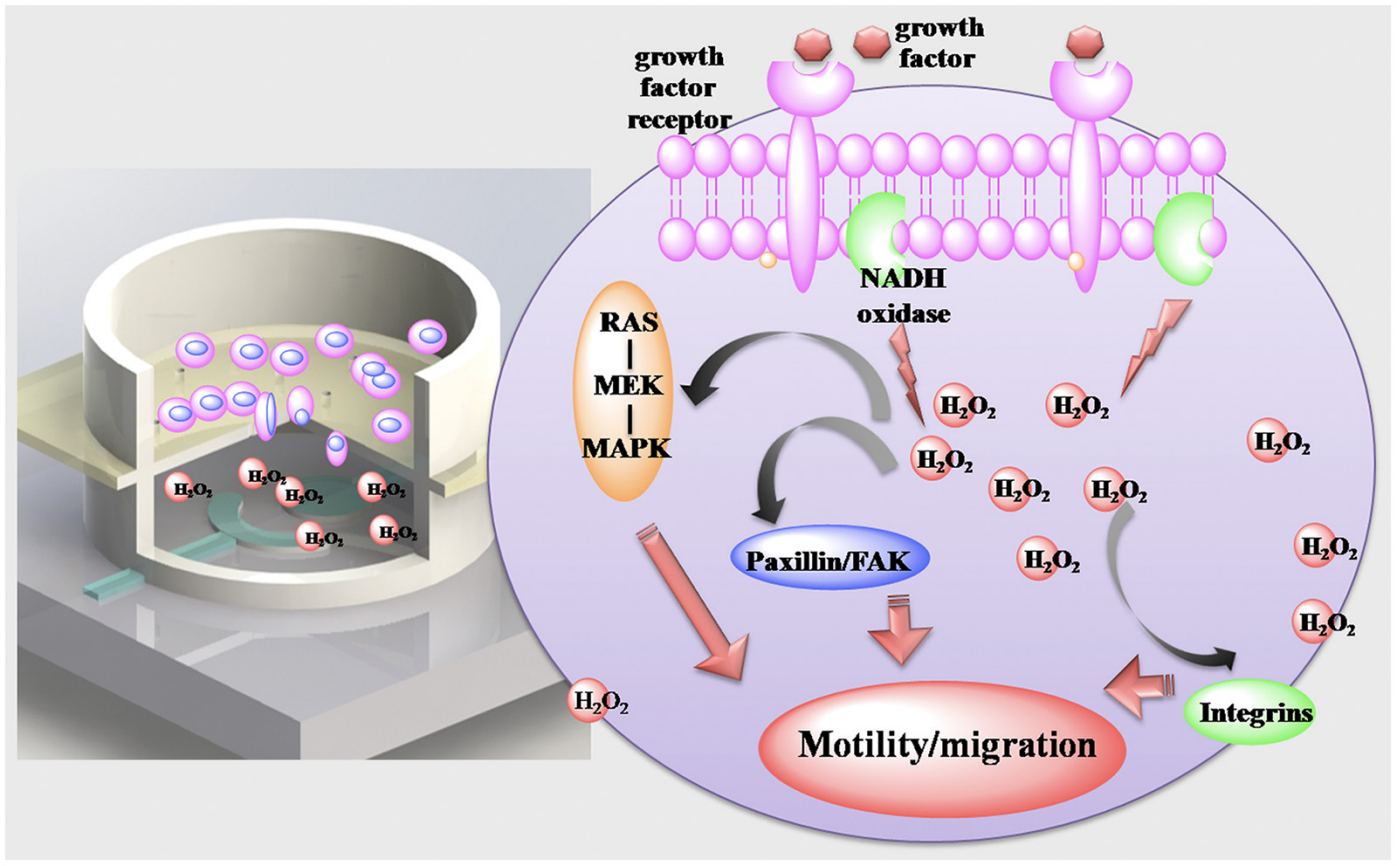
Bi-module device can *in situ* detects the generation of biochemical molecules in the process of cell migration.

As characterized in supplementary S5 Fig, CV response of serum-starved cell in serum-free RPMI 1640 and RPMI 1640 are nearly identical. While adding of serum (10% FBS) into the serum-free medium leads to a reduction peak current increasing, indicating that serum would induce production of H_2_O_2_ from serum-starved cell. The phenomena is in line with documented information that growth factors can stimulate NADPH oxidase leading to the production of H_2_O_2_.[53–55] The elevated endogenous H_2_O_2_ might trigger the activation of ERK and FAK signalling transduction pathways. The phosphorylation of ERK and FAK can lead to enhanced cell migration by activation downstream signalling proteins.[14, 53, 54] As illustrated in Fig 7, by using this device, for the first time, we quantified the H_2_O_2_ production in a trans-well cell migration setting. We anticipate the combination of electrochemical sensing with trans-well module can quantify other important biochemical molecules *in situ*, providing key information for depicting the relationship between biochemical signalling and cell function.

## Conclusions

Hydrogen peroxide is believed to modulate signalling pathways that control cell motility. However, little is known about H_2_O_2_ generation during the cell migration process. A novel bi-module device was fabricated to characterize H_2_O_2_ production *in situ* while monitoring cell migration capability. For the first time, we quantified H_2_O_2_ molecule generation from cells under a serum established chemotaxis is ~6.5×10^10^ per melanoma A375 cells, ~3.6×10^10^ per liver carcinoma Hep G2 cells and ~3.2×10^10^ per larynx carcinoma HEp-2 cells. In addition, a parallel migration assay with H_2_O_2_ generation inhibitor and decomposer demonstrated that the H_2_O_2_ generation is associated to cell migration. The bi-module cell migration platform enables *in situ* investigation for monitoring H_2_O_2_ production and cell function simultaneously, highlighting its potential for characterizing cell motility through monitoring cell secretion with rare samples and for investigation of mechanism of cell migration.

## Supporting Information

S1 Fig Cyclic voltammetric curves scanned at different time point. The electrochemical sensor was immersed in a cell culture medium for 24 h. At 0, 12, 18 and 24 h, 4 μM H_2_O_2_ was added into the cell culture medium and the cyclic voltammetric (CV) curve was recorded. Then the increase of reduction peak current (Δ current) was compared.
(TIF)

S2 Fig Amperometric performance of electrode at different potential. The amperometric response of functionalized electrode at an applied potential of -0.3, -0.4, -0.45 and -0.5V *vs* ITO reference electrode/counter electrode (RE/CE) in responding to successive addition of 4 μMH_2_O_2_ into RPMI 1640.
(TIF)

S3 Fig MTT cell growth assay. Melanoma A375 cells was seed in 96-well microplate (1×10^4^ cell per well). DPI (10 μM), catalase (5 μg mL^-1^) or DMSO (0.5%, V/V) were used to treat cells for 24 h. Then, 10 μL MTT solution was added to every well and incubated for 3 h. The purple-coloredformazan products converted by viable cells were dissolved and measured using a spectrophotometric microplate reader (ELx800t, Gene Company) at 540 nm. The experiment was performed three independent times in triplicates.
(TIF)

S4 Fig Boyden chamber migration assay. Hematoxylin and eosin staining of migrating A375 examined in a Boyden chamber assay. Different concentration of cell suspensions was seed in the upper chamber and incubated for 24 h. The results were quantified using migrating cell counted in an assay without serum in the bottom chamber as a reference.
(TIF)

S5 Fig H_2_O_2_ production from serum-starved cells by direct serum stimulation. Melanoma A375 cells were serum-starved for 8 h and then collected. RPMI 1640 medium was placed in the PDMS chamber and CV response was recorded. Then serum-starved cell (4×10^5^) was pipetted into the chamber. After 10 min, the CV response was recorded. Finally, serum (10% FBS) was added into the chamber. The CV response was recorded after 30 min incubation.
(TIF)
