# Supplementary figures and images for: Bi-Module Sensing Device to In Situ Quantitatively Detect Hydrogen Peroxide Released from Migrating Tumor Cells
Source: PLoS One. 2015 Jun 2;10(6):e0127610. doi: 10.1371/journal.pone.0127610 (PMC4452705; doi:10.1371/journal.pone.0127610)

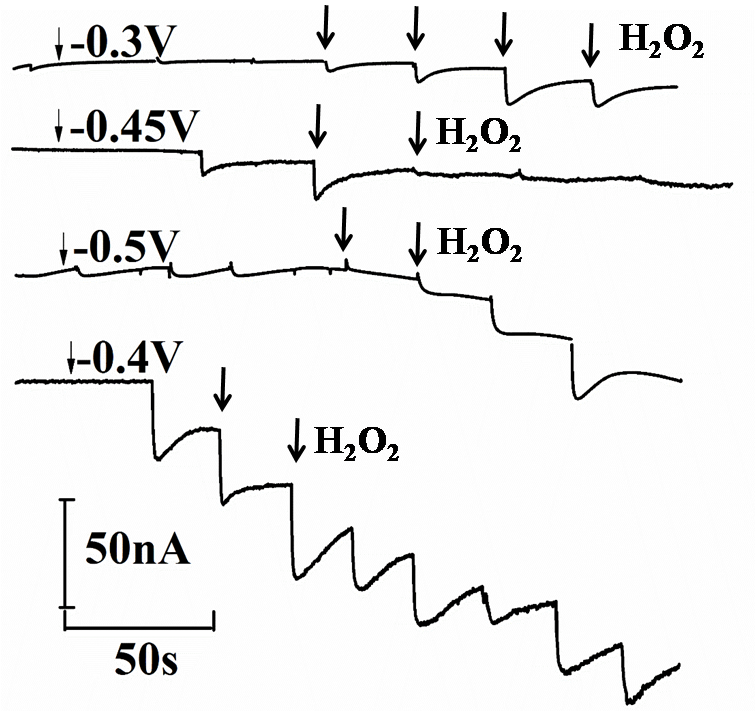

Supplement: S2 Fig — The amperometric response of functionalized electrode at an applied potential of -0.3, -0.4, -0.45 and -0.5V vs ITO reference electrode/counter electrode (RE/CE) in responding to successive addition of 4 μMH2O2 into RPMI 1640. (TIF) [file pone.0127610.s002.tif]

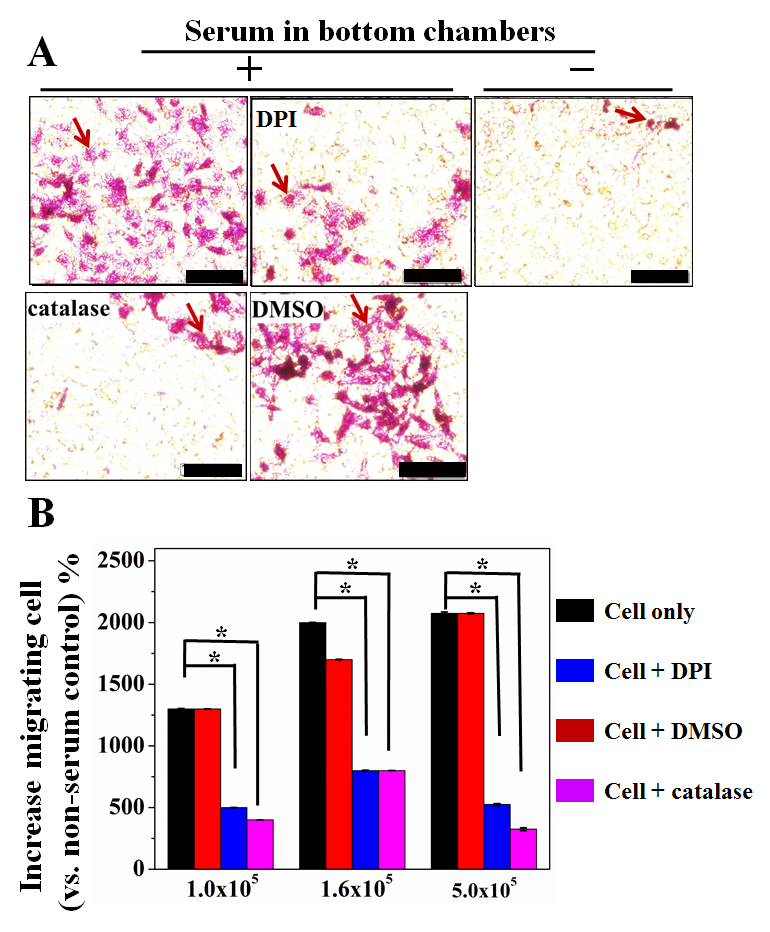

Supplement: S4 Fig — Hematoxylin and eosin staining of migrating A375 examined in a Boyden chamber assay. Different concentration of cell suspensions was seed in the upper chamber and incubated for 24 h. The results were quantified using migrating cell counted in an assay without serum in the bottom chamber as a reference. (TIF) [file pone.0127610.s004.tif]
